# A frog‐derived antimicrobial peptide as a potential anti‐biofilm agent in combating *Staphylococcus aureus* skin infection

**DOI:** 10.1111/jcmm.17785

**Published:** 2023-05-20

**Authors:** Fan Fei, Tao Wang, Yangyang Jiang, Xiaoling Chen, Chengbang Ma, Mei Zhou, Qinan Wu, Peng Cao, Jinao Duan, Tianbao Chen, James F. Burrows, Lei Wang

**Affiliations:** ^1^ Natural Drug Discovery Group, School of Pharmacy Queen's University Belfast Belfast UK; ^2^ International Joint Laboratory for Animal Tradition Chinese Medicine and Functional Peptides, College of Pharmacy Nanjing University of Chinese Medicine Nanjing China

**Keywords:** antibiofilm, brevinin‐1, membrane disruptive, Rana box, skin infections

## Abstract

*Staphylococcus aureus* (*S. aureus*), one of the most prevalent bacteria found in atopic dermatitis lesions, can induce ongoing infections and inflammation by downregulating the expression of host defence peptides in the skin. In addition, the emergence of the ‘superbug’ *Methicillin‐resistant S. aureus* (MRSA) has made the treatment of these infections more challenging. Antimicrobial peptides (AMPs), due to their potent antimicrobial activity, limited evidence of resistance development, and potential immunomodulatory effects, have gained increasing attention as potential therapeutic agents for atopic dermatitis. In this study, we report a novel AMP, brevinin‐1E‐OG9, isolated from the skin secretions of *Odorrana grahami*, which shows potent antibacterial activity, especially against *S. aureus.* Based on the characteristics of the ‘Rana Box’, we designed a set of brevinin‐1E‐OG9 analogues to explore its structure–activity relationship. Brevinin‐1E‐OG9c‐De‐NH_2_ exhibited the most potent antimicrobial efficacy in both in vitro and ex vivo studies and attenuated inflammatory responses induced by lipoteichoic acid and heat‐killed microbes. As a result, brevinin‐1E‐OG9c‐De‐NH_2_ might represent a promising candidate for the treatment of *S. aureus* skin infections.

## 1 INTRODUCTION

Atopic dermatitis (AD) (or atopic eczema), as one of the most common chronic skin diseases, is generally associated with an increased risk of bactericidal skin infection in patients and induced significant morbidity.^1^ Opportunistic pathogens, especially *Staphylococcus aureus*, colonise the skin of most AD patients and cause infections.^2^ *S. aureus* on AD lesions will secrete a set of factors to aggravate inflammation and compromise skin barrier function.^3^ Furthermore, these bacteria are commonly aggregated on epithelial surfaces and form biofilms, which impede antibiotics and host clearance mechanisms, such as antibodies and phagocytes. This makes these infections hard to treat and leads to ongoing inflammation and chronic infections.^4^, ^5^

Host defence peptides (HDPs), or antimicrobial peptides (AMPs), as one of the critical parts of the immune system, can effectively inhibit the pathogenesis of infections and protect the host from inflammation.^6^ However, the production of these peptides is often dysregulated by the microbes and inflammatory molecules present in AD lesions.^7^, ^8^, ^9^ Recently, research on human HDPs indicate they have some potential advantages for AD treatment, such as their broad spectrum antibacterial action and their immunomodulatory effects, making them a promising candidates in AD patients' management.^6^ Amphibians, found in both terrestrial and aquatic environments rich in microbes, are considered excellent natural sources of HDPs/AMPs.^10^, ^11^, ^12^ From their skin secretions, some HDPs with great pharmacological values have been identified and developed to treat skin infections. For example, Magainin, from *Xenopus laevis*, has been modified and studied for their potential to treat infected diabetic foot ulcers.^13^, ^14^ Brevinins are an influential group of AMPs from frog skin secretions, which have been found in various species of frogs, and which display potent broad spectrum antimicrobial activity, including activity against antibiotic‐resistant strains. Brevinins have also been found to display antibiofilm, anticancer, immunomodulatory and wound healing activities. These bioactivities make brevinins stand out in the crowd of the multitudinous AMPs.^15^, ^16^, ^17^, ^18^, ^19^ However, studies using brevinins against skin infections are limited. Therefore, in this study, we characterised, modified and explored the antibacterial potential of a novel brevinin‐1 AMP, brevinin‐1E‐OG9, using a porcine skin infection model. This novel peptide was identified from the skin secretions of *Odorrana grahami* and exhibited potent antibacterial properties with test pathogens, especially gram‐positive bacteria. As a characteristic part of brevinin‐1 peptides, the role of the ‘Rana Box’ has been widely discussed in previous studies, although its functions still remain elusive. In our work, we designed a set of analogues of brevinin‐1E‐OG9 by modifying the cationicity or truncating the loop structure to explore further the function of the ‘Rana Box’ in this peptide.

## 2 MATERIALS AND METHODS

### 2.1 Acquisition of skin secretions from *Odorrana grahami*

Adult *Odorrana grahami* frogs were captured in Yunnan province, PRC. Skin secretion was obtained from the dorsal skin using mild electrical stimulation. The skin secretion was collected and lyophilised before storage at −20°C. The amphibian‐related experiments in this study were reviewed by the Institutional Animal Care and Use Committee of Queen's University Belfast and approved (1st March 2011) in terms of the UK Animal (Scientific Procedures) Act 1986 (2), Project licence PPL 2694, issued by the Department of Health, Social Service and Public Safety, Northern Ireland.

### 2.2 ‘Shotgun’ cloning of the novel peptide from *Odorrana grahami* skin secretion

Brevinin‐1E‐OG9 Precursor‐Encoding cDNA was acquired from the cDNA library of *Odorrana grahami* using a NUP primer (nested universal primer; 5′‐AAGCAGTGGTATCAACGCAGAGT‐3′) and a sense primer (5′‐GAWYYARAGCCYAAADATG‐3′) (W = A or T, Y = C or T, R = A or G, D = A, G or T) that was designed to match highly conserved domains of the 5′‐untranslated regions.

### 2.3 Peptide synthesis, purification and characterisation

Brevinin‐1E‐OG9 and its analogues were synthesised through solid‐phase peptide synthesis by Tribute Peptide Synthesizer (Protein Technologies). The crude peptide was purified by employing Reverse‐Phase High‐Performance Liquid Chromatograph (RP‐HPLC; Jupiter C‐18, 250 × 10 mm, Phenomenex). Matrix‐assisted laser desorption/ionisation‐time of flight (MALDI‐TOF) was employed to measure the molecular weight of the crude and purified peptides.

### 2.4 Bioinformatics analysis

The multiple sequence alignment was performed using Clustal Omega (https://www.ebi.ac.uk/Tools/msa/clustalo/). Existing peptide sequences of all brevinin‐1 peptides were collected from UniProt database (https://www.uniprot.org/). The sequence logo was created by WebLogo 3 (http://weblogo.threeplusone.com/). The physicochemical properties of peptides were analysed using HeliQuest (https://heliquest.ipmc.cnrs.fr/cgi‐bin/ComputParams.py).^20^ The secondary structure of peptides was predicted using I‐TASSER (https://zhanggroup.org/I‐TASSER/).^21^ UCSF Chimera (version 1.15) was utilised to visualise predicted 3D models of peptides. The percentage of α‐helical degree was analysed by K2D3, (http://cbdm‐01.zdv.uni‐mainz.de/~andrade/k2d3/).^22^

### 2.5 Circular dichroism spectrum

The secondary structure of peptides was determined using a JASCO J‐815 circular dichroism (CD) spectrometer (JASCO, UK), as mentioned previously.^11^ The wavelength was set from 190 to 260 nm. The scanning speed was 200 nm/min. The bandwidth and data pitches were 1 nm and 0.5 nm, respectively. Peptides (final concentration = 50 μM) were prepared in 10 mM NH_4_AC and 50% tetrafluoroethylene (TFE; v/v, in 10 mM NH_4_AC) to mimic water and membrane‐like environments, respectively.

### 2.6 Antimicrobial activity screening

Antimicrobial assays were conducted using standard broth micro‐dilutions, as mentioned previously.^11^ Gram‐positive bacteria *S. aureus* (ATCC 6538), *Methicillin‐resistant S. aureus* (MRSA; NCTC 12493), *Enterococcus faecium* (NCTC 12697), Gram‐negative bacteria *Escherichia coli* (ATCC 8739), *Klebsiella pneumoniae* (ATCC 43816), *Pseudomonas aeruginosa* (ATCC 9027), *Acinetobacter baumannii* (ATCC BAA 747), and yeast, *Candida albicans* (ATCC 10231) were used in the test. The experiments were conducted in three independent runs.

### 2.7 Haemolytic activity

The haemolytic activity of peptides was conducted using horse erythrocytes (2%, v/v). Peptide solutions were mixed with washed horse erythrocyte suspension at 37°C for 2 h. Triton X‐100 (0.1%) was used as a positive control. The mixture was then centrifuged at 930 *g* for 5 min. The absorbance of the supernatants was detected at 570 nm. The haemolysis rate was calculated using the formula:

$$\text{Haemolysis}\;\left(\%\right)=\frac{\text{Sample group}-\text{negative group}}{\text{positive group}-\text{negative group}}\times 100\%.$$

This experiment was performed in three independent runs.

### 2.8 Kinetics time‐killing

Bacteria in the log phase were diluted using corresponding broths and mixed peptide solutions (final concentration 1, 2, 4 × MIC). At different time intervals, 10 μL of the mixture was diluted in phosphate‐buffered saline (PBS) and spotted on the agar plates. The bacterial colony number was counted after overnight incubation. The experiments were conducted in three independent runs.

### 2.9 Saline sensitivity assays

Peptide solutions were mixed with a bacterial suspension containing 150 mM NaCl, 4.5 mM KCl, 6 μM NH_4_Cl, 2 mM CaCl_2_, 1 mM MgCl_2_, 8 μM ZnCl_2_, 4 μM FeCl_3_, respectively, and incubated overnight at 37°C.^11^ MIC values of peptides under different saline environments were determined at 550 nm the next day. The experiments were conducted in three independent runs.

### 2.10 Lipoteichoic acid/lipopolysaccharide binding assay

The binding affinity of peptides to lipoteichoic acid (LTA) and lipopolysaccharide (LPS) was examined using the fluorescent dye BODIPY TR cadaverine (BC) (ThermoFisher) displacement assay. BC (5 μg/mL) was incubated with 50 μg/mL of LTA from *S. aureus* (Sigma) or LPS from *E. coli* O55:B5 (Sigma) in Tris–HCl buffer (50 mM, pH 7.4) for 4 h. Then, peptide solutions were mixed with LTA/LPS‐BC solution and incubated at 37°C for 1 h. The fluorescence was monitored at excitation 580 nm and emission 620 nm. Melittin was set as a positive control. The experiments were conducted in three independent runs.

### 2.11 Outer membrane permeability assay

*Escherichia coli* ATCC 8739 at the logarithmic phase was washed with HEPES (containing 5 mM Glucose, pH 7.4) and diluted to OD_600_ = 0.5. Peptide solutions were mixed with bacterial suspension and incubated at 37°C for 2 h. Then, the fluorescent dye N‐phenyl‐1‐naphthylamine (10 μM) was added to each well. The fluorescence was recorded at excitation 350 nm/emission 420. Melittin (10 μM) was set as a positive control. The experiments were conducted in three independent runs.

### 2.12 Cytoplasmic membrane permeability assay

The bacterial cytoplasmic membrane permeability of peptides was detected using the fluorescent dye SYTOX Green (ThermoFisher).^23^ Bacteria were incubated with peptide solutions for 2 h and the mixture was then stained with SYTOX Green stain. The fluorescence emission was measured at 523 nm with excitation at 485 nm. Melittin was used as a positive control. The experiments were conducted in three independent runs.

### 2.13 Cytoplasmic membrane potential

The impact of peptides on the bacterial membrane potential were detected using the fluorescent dye 3,3′‐dipropylthiadicarbocyanine iodide (Disc_3_ (5); ThermoFisher). Bacterial samples at the mid‐log phase were first cultured in N‐2‐hydroxyethylpiperazine‐N‐2‐ethane sulfonic acid (HEPES; containing 20 mM glucose and 0.1 M KCl, pH 7.4) for 30 min with (Disc3 (5), 0.4 μM). Bacterial suspension was then mixed with peptide solutions. The changes in fluorescence were monitored at the excitation wavelength of 620 nm and the emission wavelength of 680 nm. The experiments were conducted in three independent runs.

### 2.14 Live/Dead staining for the evaluation of the effect of peptides on bacteria

Bacteria were mixed with peptide solutions for 1 h at 37°C. After centrifuged at 3000 *g* for 15 min, bacterial cell pellets were resuspended and stained with propidium iodide (PI; Sigma) for 15 min. Unbound PI subsequently was washed away with PBS, and bacterial cells were further stained with SYTO 9 (ThermoFisher) for another 15 min. The stained bacteria were observed under Leica DMi8 fluorescence microscopy (Leica) using the 100 × oil‐immersion objective.

### 2.15 Antibiofilm assay

#### 2.15.1 Biofilm inhibition assay

Peptide solutions were first mixed with bacterial cultures (5 × 10^5^ CFU/mL) and incubated at 37°C for 24 h. The planktonic bacterial cells were gently removed by washing with PBS the next day, and methanol was utilised to fix the biofilm. Crystal violet (0.1% v/v), PBS and acetic acid (30%, v/v) were subsequently used to stain biofilm, remove remaining stains and re‐dissolve stains, respectively. The absorbance was determined at 595 nm. The experiments were conducted in three independent runs.

#### 2.15.2 Biofilm eradication assay

The bacterial culture (5 × 10^5^ CFU/mL) was cultured in a sterile 96‐well plate for 24 h to generate mature biofilms. The planktonic bacteria were removed by washing three times with sterile PBS buffer. Then, fresh broth with different peptide concentrations was added to each well and further incubated for another 24 h. Then, the plate was stained with crystal violet. The stain was solubilised in 30% acetic acid, and the absorbance was measured at 595 nm. The experiments were conducted in three independent runs.

#### 2.15.3 Anti‐persister cells assay

The antibacterial activity of peptides against persister cells was performed according to the method in James et al.'s works with minor modifications.^24^ MRSA NCTC 12493 at log phase was diluted and inoculated in a 96‐well plate. The plate was set in a shaking incubator to generate biofilm (37°C, shaking speed 220 rpm). After overnight incubation, the planktonic bacteria were gently removed by washing with PBS, and fresh medium with rifampicin (100 × MIC) was added to each well and incubated for another 24 h. After incubation, planktonic bacteria were washed with PBS again. Then, 100 μL of PBS was added to each well, and the plate was sonicated for 5 min to suspend persister cells. Peptide solutions were subsequently incubated with persister cells suspension at 37°C for 4 h. Finally, 10 μl of the mixture in each well were diluted with sterile PBS buffer and spotted on an agar plate. The number of viable persister cells was counted after overnight incubation. The experiments were conducted in three independent runs.

### 2.16 Detection of biofilm components using a fluorescent microscope

The variations of components in the MRSA NCTC 12493 biofilm stained at 1/4 and 1/2 MIC of OG9 and OG9c‐De‐NH_2_ were detected, as mentioned previously.^25^ Bacteria (5 × 10^5^ CFU/mL) were first inoculated in a 24‐well plate whose bottom of each well was covered with a sterile glass coverslip (13 mm, VMR) and treated with sub‐MIC concentrations of peptide solutions. On the next day, liquid in each well was removed, and PBS was utilised to wash to clean planktonic cells. Three fluorescent dyes, DAPI, SYPRO Ruby (ThermoFisher) and WGA‐488 (ThermoFisher), were used to stain nucleic acids, proteins and polysaccharides in EPS, respectively. The stained biofilms were observed under Leica DMi8 fluorescence microscopy (Leica) using the 20 × objective. The fluorescent intensity was determined using ImageJ (version 1.51J8, NIH).

### 2.17 MTT assay

The cytotoxicity of peptides against human keratinocyte cells (HaCaT) was studied using the MTT assay, as reported previously.^26^ HaCaT cells (1 × 10^4^ cells/mL) were seeded into a 96‐well plate with DMEM medium (Life Technologies) supplemented with 10% (v/v) foetal bovine serum (FBS), 1% (v/v) penicillin–streptomycin (PS). After that, cells were treated with a corresponding serum‐free culture medium for 4 h. Cells were treated with peptides in concentrations ranging from 25 to 100 nM. Then, 10 μL of MTT reagent (5 mg/mL) was added to each sample after overnight incubation and cells were further incubated at 37°C for another 4 h. All liquids were removed, and 100 μL of dimethyl sulfoxide was added. The absorbance of each sample was at 570 nm. The experiments were conducted in three independent runs.

### 2.18 Ex vivo skin infection model

The antibacterial efficacy of OG9 and OG9c‐De‐NH_2_ was further evaluated in an ex vivo porcine skin model, as reported.^27^ The porcine skin was shaved and cleaned with 70% ethanol and dd H_2_O. The polyethylene tubing was glued to the cleaned skin surface with cyanoacrylate glue. Exponential or persister cells were inoculated to infect each skin sample. After 15 min of incubation at 37°C, each skin sample was treated with peptides at concentrations of 1 × or 10 × MIC or vancomycin (8 μM). After another 4 h of incubation, each tubing well was washed with PBS. Ten microliters of the mixture in each well was diluted with PBS buffer and spotted on the agar plate. The number of viable bacteria cells was counted after overnight incubation. The experiments were conducted in three independent runs.

### 2.19 Reactive oxygen species detection

The anti‐inflammatory capacity of peptides was detected by measuring the production of reactive oxygen species. HaCaT cells (5 × 10^4^ cells) were seeded in a 96‐well plate and treated with peptide solutions (final concentration 1 μM) the next day. Then, 1 μg/mL of LTA or heat‐killed MRSA NCTC 12493 cells (1 × 10^6^ CFU/mL) were utilised to stimulate the cells for 24 h. After that, 2′,7′‐Dichlorofluorescein diacetate (DCFH‐DA; Sigma) was used to quantitatively detect reactive oxygen species production.^28^ The stained cells were observed under fluorescence microscopy using the 5 × objective. The fluorescent intensity was determined using ImageJ.

### 2.20 Statistical analysis

Prism (Version 9.0; GraphPad Software Inc.) was used to analyse data and generate diagrams. The standard error of the mean (SEM) for each set of data in three replicates from three experiments was shown by the error bars surrounding the mean data points. One‐way anova analysis, with multiple comparisons of the mean of each column with the mean of every other column, was used to evaluate the statistical significance (*p < 0.05, **p < 0.01, ***p < 0.001 and ****p < 0.0001).

## 3 RESULTS

### 3.1 The discovery and identification of brevinin‐1‐OG9 by ‘shotgun’ cloning

**FIGURE 1:**
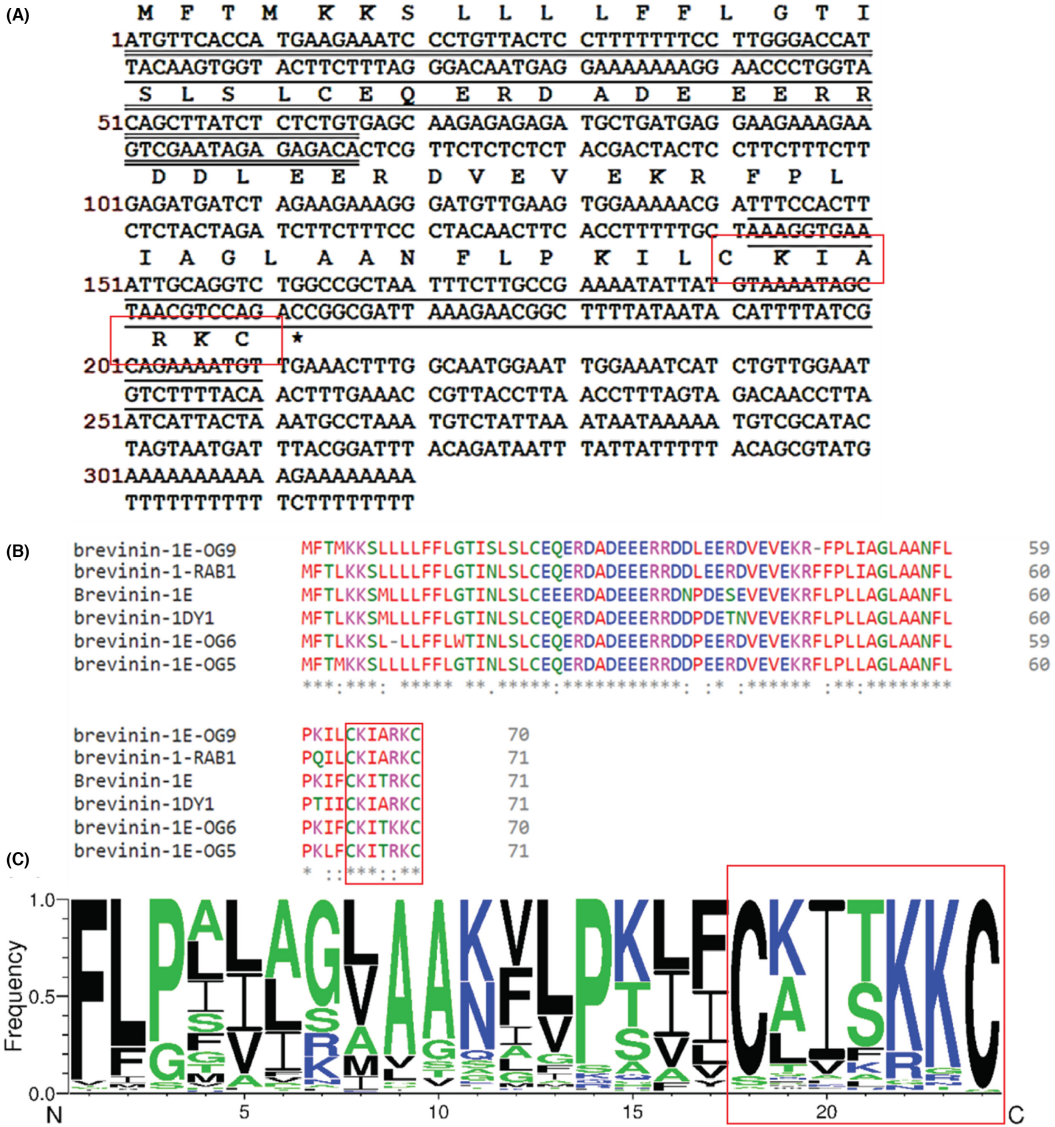
(A) The nucleotide and translated cDNA cloned from the skin secretion of *Odorrana grahami*. The putative signal peptide is double‐underlined, and the following unmarked region is the acidic ‘spacer’ region. The convertase restriction site is dot‐underlined. The mature peptide region is single underlined. The asterisk indicates the position of the stop codon; (B) The alignment of the sequence of OG9 with other similar peptides. The red letters mean nonpolar amino acids with hydrophobic residues; the green letters mean polar amino acids with hydrophilic residues; the blue letters mean acidic amino acids with negatively charged; the pink letters mean essential amino acids with positively charged. The number at the end of each line represents the number of amino acids in the sequence. The ‘.’ and ‘:’, respectively, indicate the low and high similarity of amino acid residues in each row. The asterisks indicate the identical amino acid residues in each sequence; (C) Frequency of existing amino acids in all the brevinin‐1 peptides based on the UniProt database. The ‘Rana‐Box’ was shown in red.

The complete cDNA encoding the biosynthetic precursor of a novel peptide was cloned from the skin secretion‐derived cDNA library of *Odorrana grahami* (Figure 1A). The translated open reading frame consists of 70 amino acid residues, which contains three domains, including the highly preserved putative signal peptide region, the acidic ‘spacer’ region with rich, acidic amino acid residues, a Lys (K)–Arg (R) convertase restriction site, and mature peptide region with 23 amino acids residues. Alignment results indicate that this novel peptide shows a high degree of similarity with some reported brevinin‐1 peptides and it shared 95.65% homology with brevinin‐1‐RAB1. The results demonstrated that this novel peptide belongs to the brevinin‐1 family, and it was named brevinin‐1E‐OG9 (abbreviated as OG9) (Figure 1B). The nucleotide sequence of OG9 was uploaded in the GenBank with an accession number of OQ718811.

### 3.2 Database‐based peptide design and characterisation of OG9 and its analogues

Brevinin‐1 peptides display broad spectrum antimicrobial activity and has been speculated that this is due to the interaction of their α‐helical structures with the anionic lipid bilayers of bacterial membranes.^18^ The significant role of the ‘Rana Box’ in the brevinin‐1 family and other peptide families has been examined previously.^29^ However, the role of the cationic residues, especially in the ‘Rana Box’, has not been examined, although the positively charged residues are thought to play a significant role in attracting the anionic charged bacteria cell membranes.^30^ Therefore, here we examined the cationic residues of the ‘Rana Box’ in the following function–structure relationship studies. The exploration of cationic residues in the ‘Rana Box’ was based on the frequency of residues in all brevinin‐1 sequences in the Uniport database. The amino acid frequency results aided in the selection of second‐best residues or in avoiding changes to conserved sites.

First, the ‘Rana Box’ at the C‐terminal end was truncated in OG9‐De to assess its effects on antimicrobial activity. OG9‐De‐NH_2_ was amidated at C‐terminus of OG9‐De. OG9a was modified by substituting Lys_18_ with Ala_18_ to investigate the relationship between positive charges within the ‘Rana Box’ and the antimicrobial activity of this peptide without changing the conserved residue Lys_22_. The reason for choosing Ala was that Ala was the second most frequent residue at position 18 (Figure 1C). This modification decreased the total net charge of OG9 from +4 to +3 to assess the antimicrobial activity in relation to the number of positive charges. OG9b was designed by substituting Ala_8_ with Lys to OG9a to keep the same net charges with parent peptide OG9. Based on the above, according to the HeliQuest results of OG9, a unique arrangement of positively charged residues lining up in a row was observed, which was also the design principle of OG9c, except for lining up the highly conserved residue Lys_22_. In the peptide OG9c‐De‐NH_2_, the ‘Rana Box’ was removed from OG9c‐De and the peptide was amidated at the C‐terminus to maintain the same net charge as the parent peptide (Table 1). The molecular mass weight of synthetic peptides was confirmed by MALDI‐TOF MS. The measured mass weights were consistent with theoretical values, indicating the synthesis and purification were successful (Figure S1).

**TABLE 1:** Partial physicochemical properties of OG9 and its analogues.

Peptide	Sequence	Theoretical MW	Net charge	Hydrophobicity	*μ*Hrel^a^ <*μ*H>
OG9	FPLIAGLAANFLPKILCKIARKC	2499	4	0.737	0.355
OG9a	FPLIAGLAANFLPKILCAIARKC	2442	3	0.794	0.336
OG9b	FPLIAGLKANFLPKILCAIARKC	2499	4	0.737	0.307
OG9c	FPLIARLAAKFLPKILCGIANKC	2499	4	0.737	0.372
OG9c‐De	FPLIARLAAKFLPKIL	1811	3	0.835	0.470
OG9c‐De‐NH_2_	FPLIARLAAKFLPKIL‐NH_2_	1810	4	0.835	0.470
OG9‐De	FPLIAGLAANFLPKIL	1698	1	0.923	0.388
OG9‐De‐NH_2_	FPLIAGLAANFLPKIL‐NH_2_	1697	2	0.923	0.388

^a^ The relative hydrophobic moment (*μ*Hrel).

### 3.3 Conformational analysis of OG9 and its analogues

**FIGURE 2:**
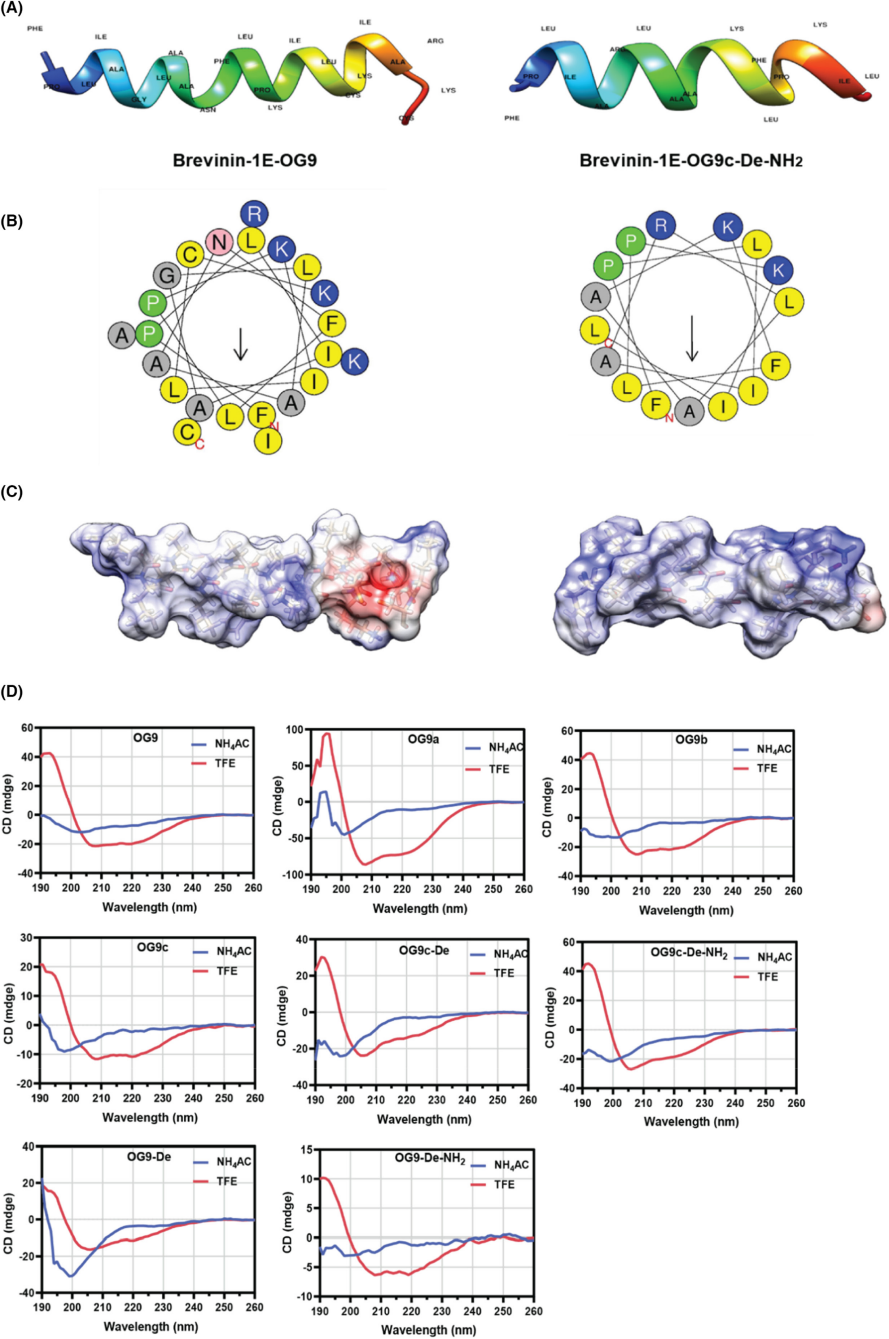
Characterisation of OG9 and OG9c‐De‐NH_2_. (A) Three‐dimensional models of OG9 and OG9c‐De‐NH_2_; (B) helical‐wheel projections of peptides analysed using HeliQuest server. Amino acids with a positive charge are shown in blue, while yellow represents hydrophobic amino acids. Alanine and glycine are in grey, proline is in proline and glutamine is pink; (C) Electrostatic potential surface of peptide OG9 (left) and OG9c‐De‐NH_2_ (right); (D) CD spectra of OG9 and its analogues in 10 mM NH_4_AC solution and 50% TFE/10 mM NH_4_AC solution.

The prediction results showed that most analogues inherited the alpha‐helical structure from the parent peptide, OG9 (Figure 2A–C and Figure S2). CD results further confirmed that all analogues adopted an alpha‐helical structure in 50% TFE solution, except for OG9‐De and OG9‐De‐NH_2_, which had fewer positive charges compared with other analogues (Figure 2D). The α‐helical degrees of OG9 and its analogues were predicted with K2D3 online server (Table 2).

**TABLE 2:** Estimated α‐helical degrees of OG9 and its analogues in 10 mM NH_4_AC solution and 50% TFE/10 mM NH_4_AC solution.

Peptide	OG9	OG9a	OG9b	OG9c	OG9c‐De	OG9c‐De‐NH_2_	OG9‐De	OG9‐De‐NH_2_
10 mM NH_4_Ac	23.05%	40.8%	2.8%	11.25%	2.52%	8.24%	19.55%	2.95%
50% TFE/10 mM NH_4_Ac	95.37%	95.44%	95.36%	83.37%	93.57%	95.12%	37.96%	55.7%

### 3.4 Haemolysis of OG9 and its analogues

**FIGURE 3:**
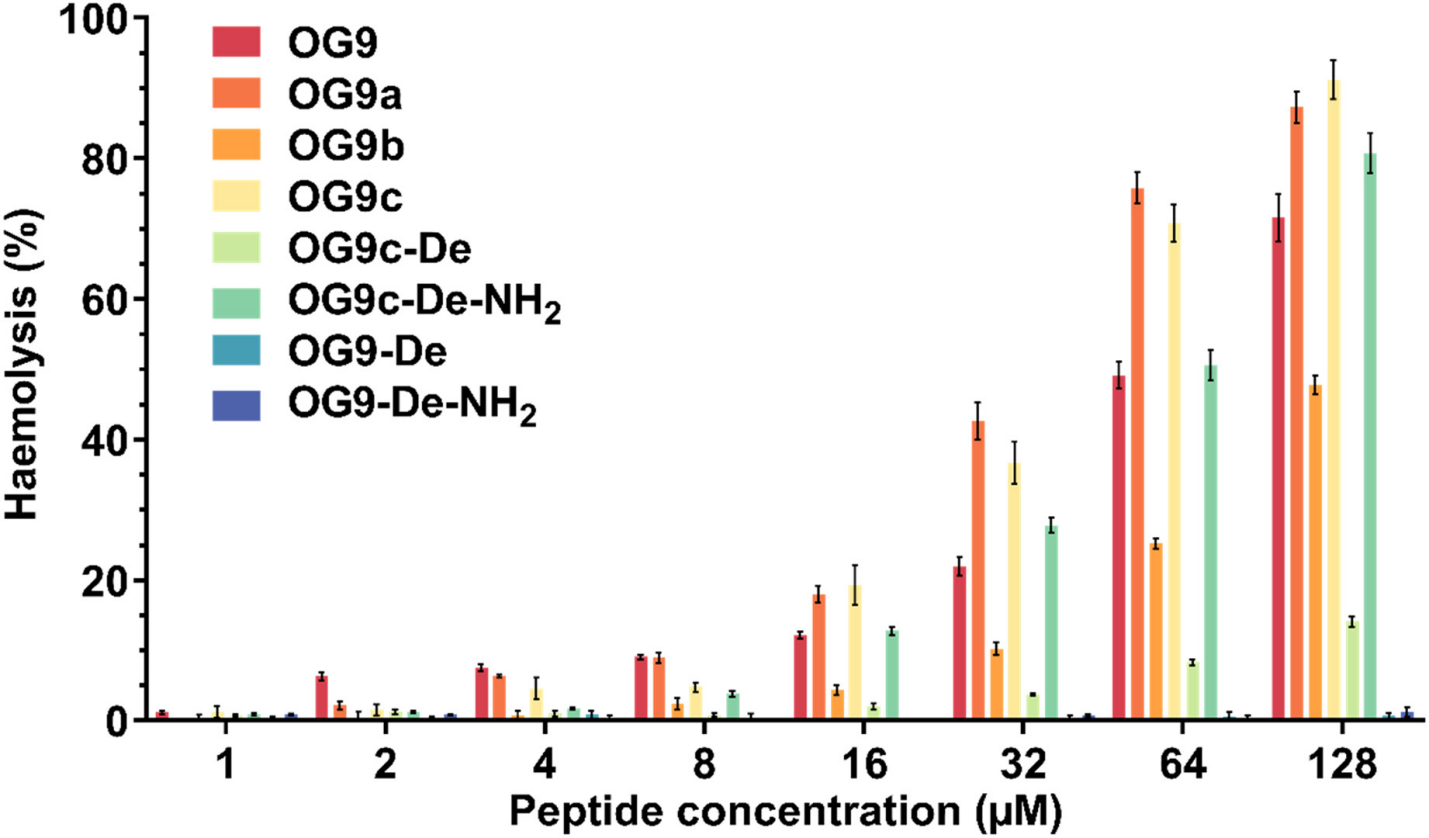
Haemolysis of OG9 and its analogues against horse erythrocytes. Values are the means ± SEM for three independent experiments.

OG9 shows obvious haemolytic activity in a concentration‐dependent manner (Figure 3). The haemolysis reaches a peak (around 85%) at the highest test concentration (128 μM). The haemolysis induced by OG9‐De and OG9‐De‐NH_2_ was greatly decreased, and no apparent haemolysis was observed even at 128 μM. Replacing Lys_18_ with Ala_18_ made no noticeable impact on the haemolysis of OG9a. The haemolytic activity of OG9a is even slightly increased compared to the parent peptide. Based on OG9a, replacing Ala_8_ with Lys_8_ greatly decreased the haemolysis activity of OG9b. The highest haemolysis rate of OG9b at 128 μM declined from 90% to 50%. Moving two positively charged resides out the ‘Rana‐Box’ increased the haemolytic activity of OG9c. Like OG9‐De, the removal of ‘Rana Box’ also helps to control the haemolysis of the OG9c‐De. However, the amidation of OG9c‐De improved the haemolytic activity of OG9c‐De‐NH_2_, especially when the concentration was above 64 μM.

### 3.5 The screening of antimicrobial activity of OG9 and its analogues

OG9 showed a broad spectrum antibacterial activity, but no antifungal activity. The truncation of the ‘Rana Box’ entirely extinguished the antimicrobial activity of the parent peptide, as well as the amidated one. OG9a slightly decreased its antimicrobial activity when the net charge was reduced by one, especially towards gram‐negative bacteria, and it lost all activity against *P. aeruginosa* ATCC 9027. The antimicrobial activity of OG9b was poorer, with a total loss of activity against the drug‐resistant gram‐positive bacteria, although it was better than OG9a. The antimicrobial activity of OG9c was increased, and it even showed antifungal activity. The truncation of the ‘Rana Box’ for OG9c turned out to be interesting because the antimicrobial activity of OG9c‐De decreased compared with that of OG9, like that of OG9‐De, while the antifungal activity increased fourfold. The amidated OG9c‐De, OG9c‐De‐NH_2,_ showed the best antimicrobial activity among all the analogues and kept the antifungal activity seen in OG9c‐De (Table 3). The geometric means of MICs of OG9c‐De‐NH_2_ against test bacteria was 3.6 μM, much lower than any other analogues. Considering its negligible haemolysis at antibacterial concentrations and the most potent antibacterial activities, OG9c‐De‐NH_2_ was selected for further study.

**TABLE 3:** Minimum inhibitory concentrations (MICs) (μM)/Minimum bactericidal concentrations (MBCs) (μM) of peptides against selected pathogens and geometric means (GM) of MICs of Grams bacteria.

Org.	Gram‐positive (+) (μM)			Gram‐negative (−) (μM)			GM MIC (μM)	Fungus (μM)
Peptide	*S. aureus* ATCC 6538	MRSA NCTC 12493	*E. faecalis* NCTC 12697	*E. coli* ATCC 8739	*K. pneumoniae* ATCC 43816	*P. aeruginosa* ATCC 9027	G+/G−/Bacteria/total bacteria	*C. albicans* ATCC 10231
OG9	4/8	16/32	16/16	4/4	64/64	16/16	10.1/16/12.7	>128/>128
OG9a	4/8	16/16	16/16	8/8	16/16	>128/>128	10.1/40.3/20.2	>128/>128
OG9b	64/128	>128/>128	>128/>128	8/8	16/16	32/64	161.3/16/50.8	>128/>128
OG9c	4/4	8/8	16/16	4/4	8/8	16/16	8/8/8	64/64
OG9c‐De	16/16	16/16	64/64	16/16	32/32	16/64	25.4/20.2/22.6	16/16
OG9c‐De‐NH_2_	2/2	2/2	8/8	2/2	4/4	8/8	3.2/4/3.6	16/16
OG9‐De	>128/>128	>128/>128	>128/>128	>128/>128	>128/>128	>128/>128	—	>128/>128
OG9‐De‐NH_2_	>128/>128	>128/>128	>128/>128	>128/>128	>128/>128	>128/>128	—	>128/>128

### 3.6 Kinetic time‐killing of OG9 and OG9c‐De‐NH_2_

**FIGURE 4:**
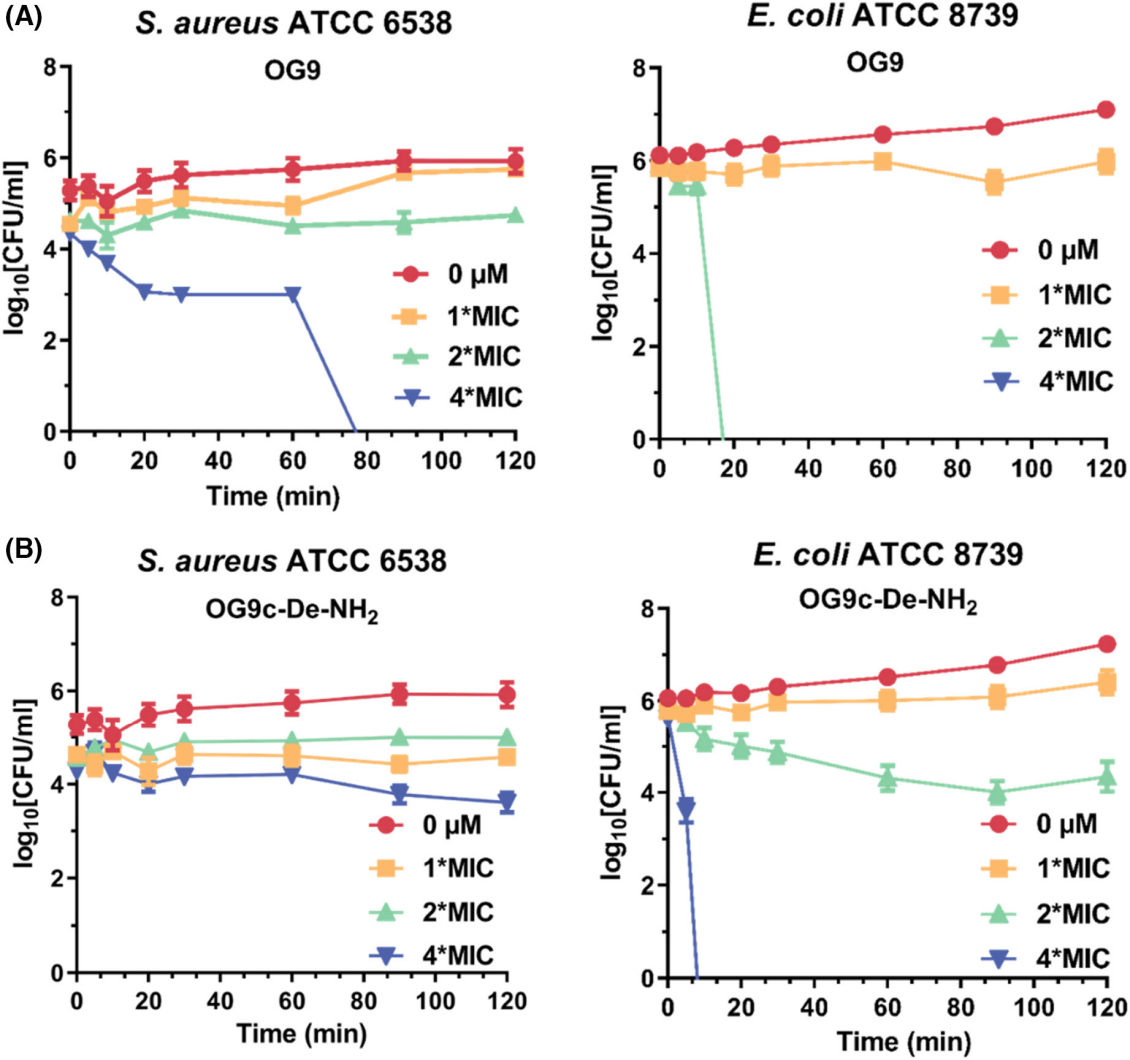
Kinetic time‐killing of (A) OG9 and (B) OG9c‐De‐NH_2_ against *Staphylococcus aureus* ATCC 6538 and *Escherichia coli* ATCC 8739. Data from three independent experiments, each in triplicate, are represented as mean ± SEM.

To access killing rate of OG9 and OG9c‐De‐NH_2_, the kinetic time‐killing assay was performed. OG9 and OG9c‐De‐NH_2_ showed bacteriostatic activity against both *S. aureus* ATCC 6538 and *E. coli* ATCC 8739 at their MIC concentrations out to 120 min (Figure 4). As for *S. aureus* ATCC 6538, even at 4 × MIC, OG9c‐De‐NH_2_ only inhibited the growth of bacteria. OG9 also had an inhibitory effect at twofold MIC while eradicating *S. aureus* ATCC 6538 after treatment for 90 min at 4 × MIC. Regarding *E. coli* ATCC 8739, OG9 had a quicker elimination rate than *S. aureus* ATCC 6538, with the elimination of bacteria after 20 min at 2 × MIC and instant effect at 4 × MIC. Following a similar pattern, OG9c‐De‐NH_2_ had no killing effect against *E. coli* ATCC 8739 at 2 × MIC while being bactericidal was observed at 4 × MIC after 10 min treatment.

### 3.7 Saline tolerance of OG9 and OG9c‐De‐NH_2_

To assess the tolerance of peptides under the background of salt solutions which mimicked the human body fluid environment, several salt solutions were selected as follows. Among test solutions, 150 mM NaCl had the most marked impact upon antimicrobial activity, causing the MIC to rise fourfold, except for the OG9c‐De‐NH_2_ against *S. aureus* ATCC 6538 where a slight increase in MBC from 2 to 4 μM was observed. Besides that, 2 mM CaCl_2_ solution appeared to have a greater impact on the antimicrobial activity against *E. coli* ATCC 8739, showing a 16‐fold increase in MIC of both peptides. OG9c‐De‐NH_2_ generally had better salt solution tolerance than OG9, with the MIC being nearly unchanged, especially against *S. aureus* ATCC 6538 (Table 4).

**TABLE 4:** MICs (μM)/MBCs (μM) of peptides against *Staphylococcus aureus* ATCC 6538 and *Escherichia coli* ATCC 8739 in different salt solutions.

Org.	Peptide	Normal	150 mM NaCl	4.5 mM KCl	6 μM NH_4_Cl	2 mM CaCl_2_	1 mM MgCl_2_	8 μM ZnCl_2_	4 μM FeCl_3_
*S. aureus* ATCC 6538	OG9	4/8	16/16	8/8	8/8	8/8	8/8	8/8	8/8
	OG9c‐De‐NH_2_	2/2	2/4	2/4	2/2	2/2	2/4	2/2	2/2
*E. coli* ATCC 8739	OG9	4/4	16/16	4/4	4/4	64/64	8/8	4/4	4/4
	OG9c‐De‐NH_2_	2/2	8/8	2/2	2/2	32/64	4/4	2/2	2/2

### 3.8 Preliminary mechanistic studies of OG9 and OG9c‐De‐NH_2_

Calcium (Ca^2+^) ions, one of the critical factors in guaranteeing the stabilisation of bacterial cell walls, or Gram‐negative bacterial outer membrane, produced a noticeable impact on the antibacterial performance of AMPs.^31^, ^32^ Therefore, we presumed that OG9 and OG9c‐De‐NH_2_ might show disruptive effects on the bacterial membrane. To study the potential antibacterial mechanisms of OG9 and OG9c‐De‐NH_2_, we conducted a set of assays to explore the impact of peptides on bacterial membranes.

**FIGURE 5:**
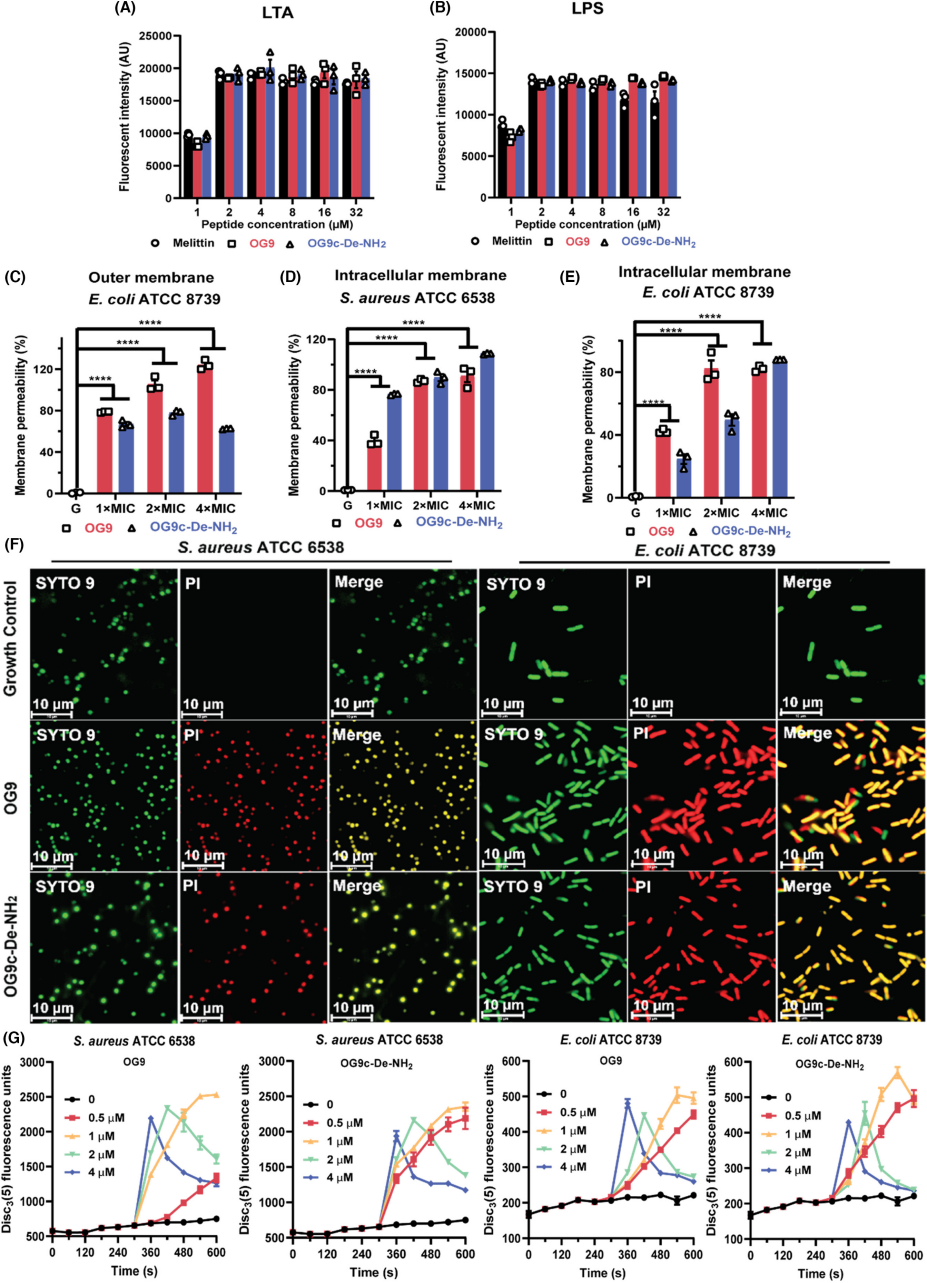
(A, B) Ability of OG9, OG9c‐De‐NH_2_ and melittin binding of lipoteichoic acid (LTA) and lipopolysaccharide; (C) Outer membrane permeability induced by OG9 and OG9c‐De‐NH_2_; (D, E) Effects of OG9 and OG9c‐De‐NH_2_ on the inner membrane permeability of *Staphylococcus aureus* ATCC 6538 and *Escherichia coli* ATCC 8739, detected using SYTOX Green dye; (F) Fluorescence microscopic images of *S. aureus* ATCC 6538 and *E. coli* ATCC 8739 treated with OG9 and OG9c‐De‐NH_2_ at 2× MIC. The scale bar is 10 μm; (G) Cytoplasmic membrane depolarisation of *S. aureus* ATCC 6538 and *E. coli* ATCC 8739, monitored using membrane potential dye Disc_3_ (5). The data shown are the means ± SEM of three independent experiments using three replicates. The significance is indicated by ****p < 0.0001.

First, we evaluated the binding affinity of OG9 and OG9c‐De‐NH_2_ with LTA and LPS using a fluorescent dye BC‐based displacement assay. OG9 and OG9c‐De‐NH_2_ showed obvious LTA and LPS‐binding activity in a concentration‐dependent manner (Figure 5A,B). Based on this result, we further evaluated the possible disruptive effects of these two peptides on the outer or/and intracellular membrane of bacteria. OG9 showed an outer membrane‐disruptive impact in a concentration‐dependent manner, while the permeability rate of OG9c‐De‐NH_2_ was kept at around 65% under different test concentrations (Figure 5C). Subsequently, the impact of peptides on the intracellular membrane of bacteria was further evaluated with a SYTOX Green staining assay. Both test peptides showed concentration‐dependent effects on the intracellular membrane of *S. aureus* ATCC 6538 and *E. coli* ATCC 8739 (Figure 5D,E). The permeability rates of OG9 and OG9c‐De‐NH_2_ stayed around 90%, indicating their striking effect to the bacterial intracellular membranes. SYTO9/PI staining results further proved the high membrane permeabilisation induced by OG9 and OG9c‐De‐NH_2_ (Figure 5F). The electric potential (ΔΨ), as one of the essential parts of bacterial transmembrane potentials, plays a critical role in fundamental cellular functions.^33^ Results shown in Figure 5G indicated that the treatment of OG9 and OG9c‐De‐NH_2_ remarkably increased fluorescence in *S. aureus* ATCC 6538 and *E. coli* ATCC 8739, demonstrating the apparent damage of peptides on the ΔΨ of the membrane transmembrane potentials.

### 3.9 Antibiofilm activity of OG9c‐De‐NH_2_

**FIGURE 6:**
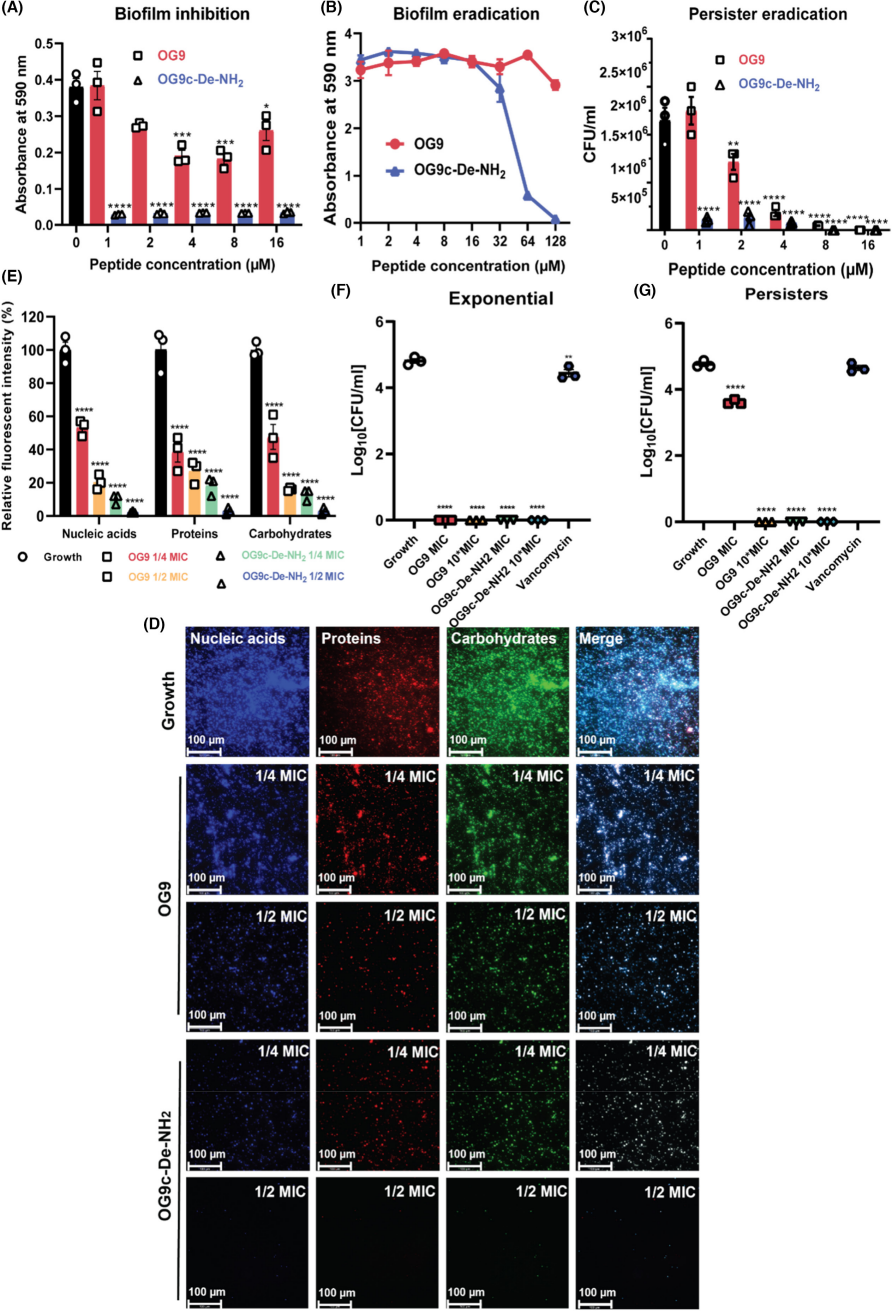
(A) Effects of OG9 and OG9c‐De‐NH_2_ on biofilm‐formation; (B) Biofilm eradication of peptides on the mature biofilm; (C) Anti‐persister effects on MRSA NCTC 12493 persister cells; (D) Effects of OG9 and OG9c‐De‐NH_2_ on the matrix component level of MRSA NCTC 12493. Three fluorescent dyes (DAPI, SYPRO Ruby and WGA‐488) were utilised, respectively, to stain nucleic acids (blue), proteins (red) and carbohydrates (green). The scale bar is 100 μM. (E) Relative fluorescent intensity of each detected component under treated or untreated conditions. (F, G) Efficacy of OG9 and OG9c‐De‐NH_2_ in an ex vivo porcine skin infection model infected with MRSA NCTC 12493. The data are derived from three independent experiments and shown as the means ± SEM. The significance is indicated by ****p < 0.0001, ***p < 0.001, **p < 0.01 and *p < 0.05.

OG9 and G9c‐De‐NH_2_ showed apparent inhibitory effects on biofilm formation using test concentration ranges. However, for their mature biofilm‐eradication activities, their MBEC values were higher than their MBIC values (Table S1, Figure 6A,B). It is worth noting that MBIC values of OG9c‐De‐NH_2_ against *S. aureus* ATCC 6538 and MRSA NCTC 12493 were 1 μM, which were lower than its MICs (both 2 μM), indicating OG9c‐De‐NH_2_ showed potent antibiofilm activity against these two bacteria strains. OG9c‐De‐NH_2_ showed potent anti‐persister effects compared to OG9, and most persisters were eradicated at 1 μM (1/2 MIC) (Figure 6C). The effects of OG9 and OG9c‐De‐NH_2_ on extracellular DNA (eDNA), protein and polysaccharide formation in MRSA NCTC 12493 biofilms were also investigated. As shown in Figure 6D,E, the presence of sub‐MIC concentrations of OG9 and OG9c‐De‐NH_2_ markedly reduced the levels of these compounds compared to untreated growth controls. In particular, 1/2 MIC OG9c‐De‐NH_2_ (1 μM) resulted in a near complete loss of these three components, indicating its potent antibiofilm capacity.

### 3.10 Ex vivo antibacterial activity of OG9 and OG9c‐De‐NH_2_

As shown in Figure 6F,G, OG9 and OG9c‐De‐NH_2_ showed significant anti‐MRSA properties against both exponential MRSA cells and persisters. Vancomycin, however, merely showed a weak inhibiting effect on the growth of exponential cells. At the concentration of 10 × MIC, both peptides eradicate MRSA. However, at the MIC, OG9 merely showed bacteriostatic effects on MRSA NCTC 12493 persisters, whereas OG9c‐De‐NH_2_ still eliminated all persisters, indicating more potent antibacterial activity of OG9c‐De‐NH_2_.

### 3.11 Inhibition on inflammation response in human keratinocytes HaCaT cells

**FIGURE 7:**
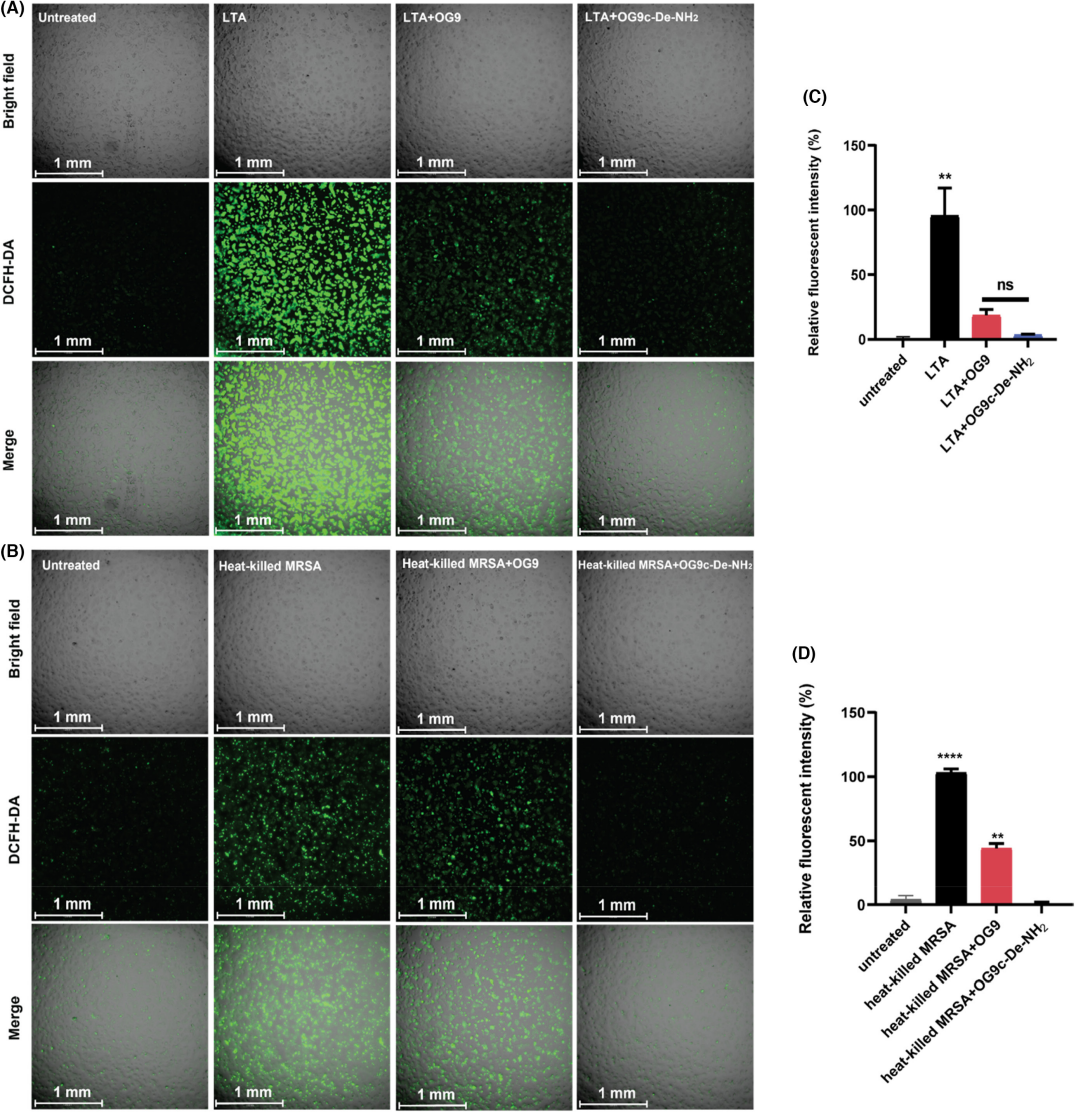
Effects of OG9 and OG9c‐De‐NH_2_ on the production of ROS in (A) LTA/(B) heat‐killed MRSA‐stimulated human keratinocytes HaCaT cells. The scale bar is 1 mm. (C, D) Relative fluorescent intensity of detected ROS under treated or untreated conditions. The significance is indicated by ****p < 0.0001 and **p < 0.01.

High numbers of *S. aureus* in skin of AD patients often induce skin inflammation. As key signalling indicators, ROS play a critical role in the pathogenesis of inflammation. Herein, we employed human keratinocytes HaCaT cells and measured the effects of peptides on the production of ROS in the response of LTA and heat‐killed MRSA. OG9c‐De‐NH_2_ showed relatively weaker cytotoxicity (IC_50_ = 69.6 μM) against HaCaT cells than its parent peptide (IC_50_ = 9.8 μM; Figure S3). OG9c‐De‐NH_2_ exhibited more potent inhibitory effects on the production of ROS induced by LTA or heat‐killed microbe compared to the parent peptide (Figure 7).

## 4 DISCUSSION

Antimicrobial peptides, due to their potent antimicrobial capacity and low possibility of developing resistance, have gained increasing attention and been given great expectations as novel antibacterial agents to cover the shortage of effective antibiotics.^34^, ^35^ Herein, we identified a novel brevinin‐1 type AMP, OG9, and designed a set of analogues to study its structure–activity relationship. OG9c‐De‐NH_2_, which had the most potent antibacterial activity, was selected to further study the potential of these peptides for use in *S. aureus*‐induced skin infections.

Modification of the OG9 ‘Rana Box’ indicates that the loop structure does play an important role in the antibacterial activity of this peptide, and this is more likely to be contributed by the cationic residues within the loop, since the truncation of the ‘Rana Box’ after removing two cationic residues out did not deprive the antibacterial activity of OG9c‐De and OG9c‐De‐NH_2_. The haemolysis results indicate that it might be the loop structure, rather than the cationic residues inside the ‘Box’ that affect the haemolytic activity of brevinin‐1 s.

OG9c‐De‐NH_2_ showed potent binding with LTA and LPS and can significantly induce membrane permeabilisation of bacteria. However, given the low killing rate, OG9c‐De‐NH_2_ might not kill bacteria via rapid lysis, although its ability to cause membrane permeability may contribute to antimicrobial efficacy. In addition, OG9c‐De‐NH_2_ exhibited potent antibiofilm activity and inhibitory effect on ROS production stimulated by LTA or heat‐killed microbe. Overall, our studies identified a novel AMP and developed an analogue with potential as an antimicrobial agent.

## AUTHOR CONTRIBUTIONS

**Fan Fei:** Conceptualization (lead); data curation (equal); formal analysis (equal); investigation (equal); writing – original draft (equal). **Tao Wang:** Data curation (equal); formal analysis (equal); investigation (equal); methodology (equal); writing – original draft (equal). **Yangyang Jiang:** Investigation (equal); methodology (equal). **Xiaoling Chen:** Formal analysis (supporting). **Chengbang Ma:** Resources (equal). **Mei Zhou:** Methodology (supporting); writing – review and editing (equal). **Qinan Wu:** Resources (equal). **Peng Cao:** Resources (equal). **Jinao Duan:** Resources (equal). **Tianbao Chen:** Methodology (supporting). **James F. Burrows:** Writing – review and editing (equal). **Lei Wang:** Methodology (supporting); writing – review and editing (equal).

## CONFLICT OF INTEREST STATEMENT

The authors declare no conflict of interest.

## Supporting information


Appendix S1.

## Data Availability

The data that support the findings of this study are available from the corresponding author upon reasonable request.
