# Supplementary material for: A frog‐derived antimicrobial peptide as a potential anti‐biofilm agent in combating Staphylococcus aureus skin infection
Source: J Cell Mol Med. 2023 May 20;27(11):1565–79. doi: 10.1111/jcmm.17785 (PMC10243163; doi:10.1111/jcmm.17785)
Supplement: Supplementary file 1 — Appendix S1. [file JCMM-27-1565-s001.docx]

**Supplementary Material**

**A Frog-derived antimicrobial peptide as a potential anti-biofilm agent in combating *Staphylococcus aureus* skin infection**

**Fan *et al*.**

OG9

OG9a


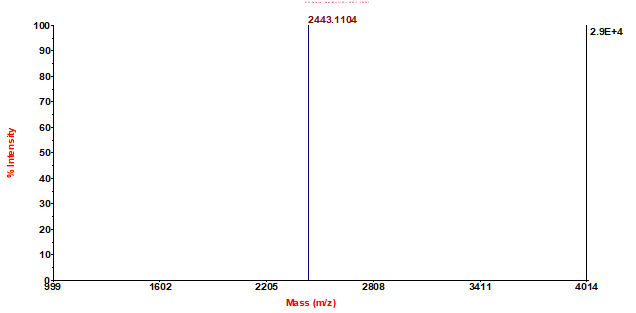


OG9b


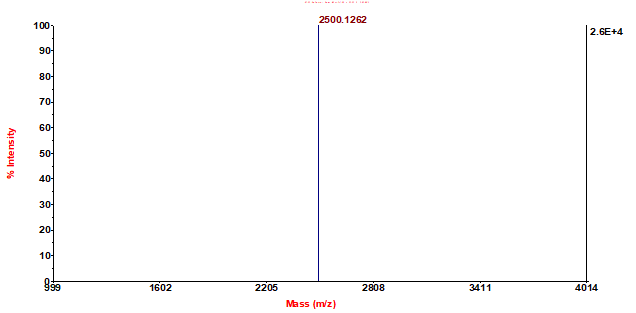


OG9c


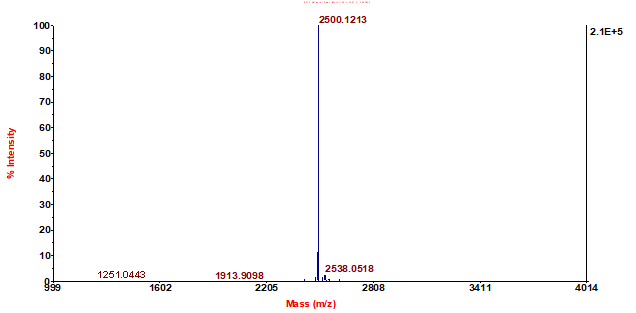


OG9c-De


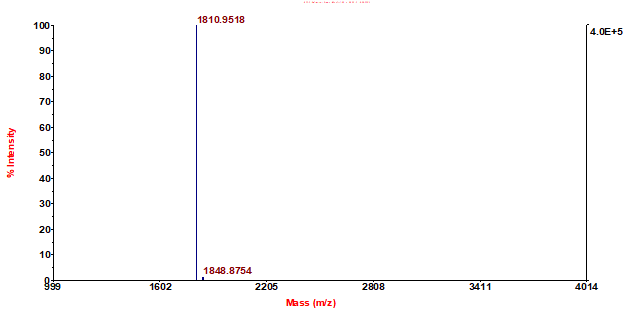


OG9c-De-NH_2_

OG9-De


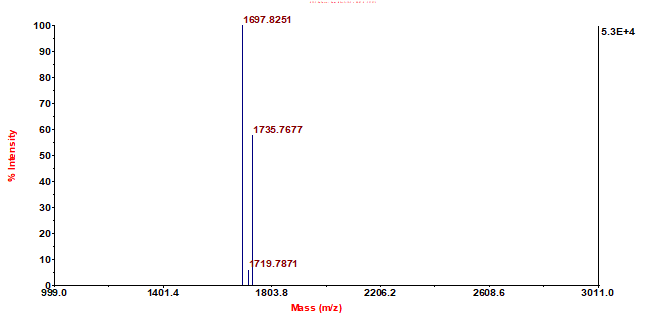


OG9-De-NH_2_


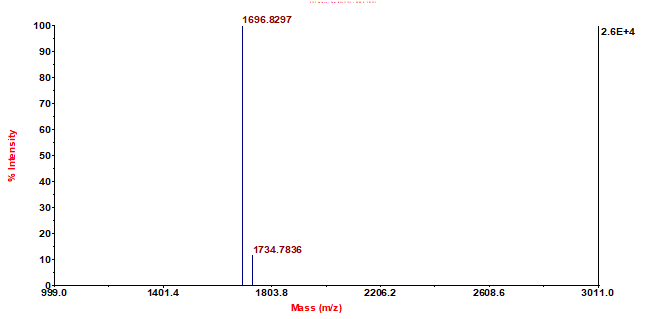


**Figure S1.** MALDI-TOF mass spectrum of OG9 and its analogues.


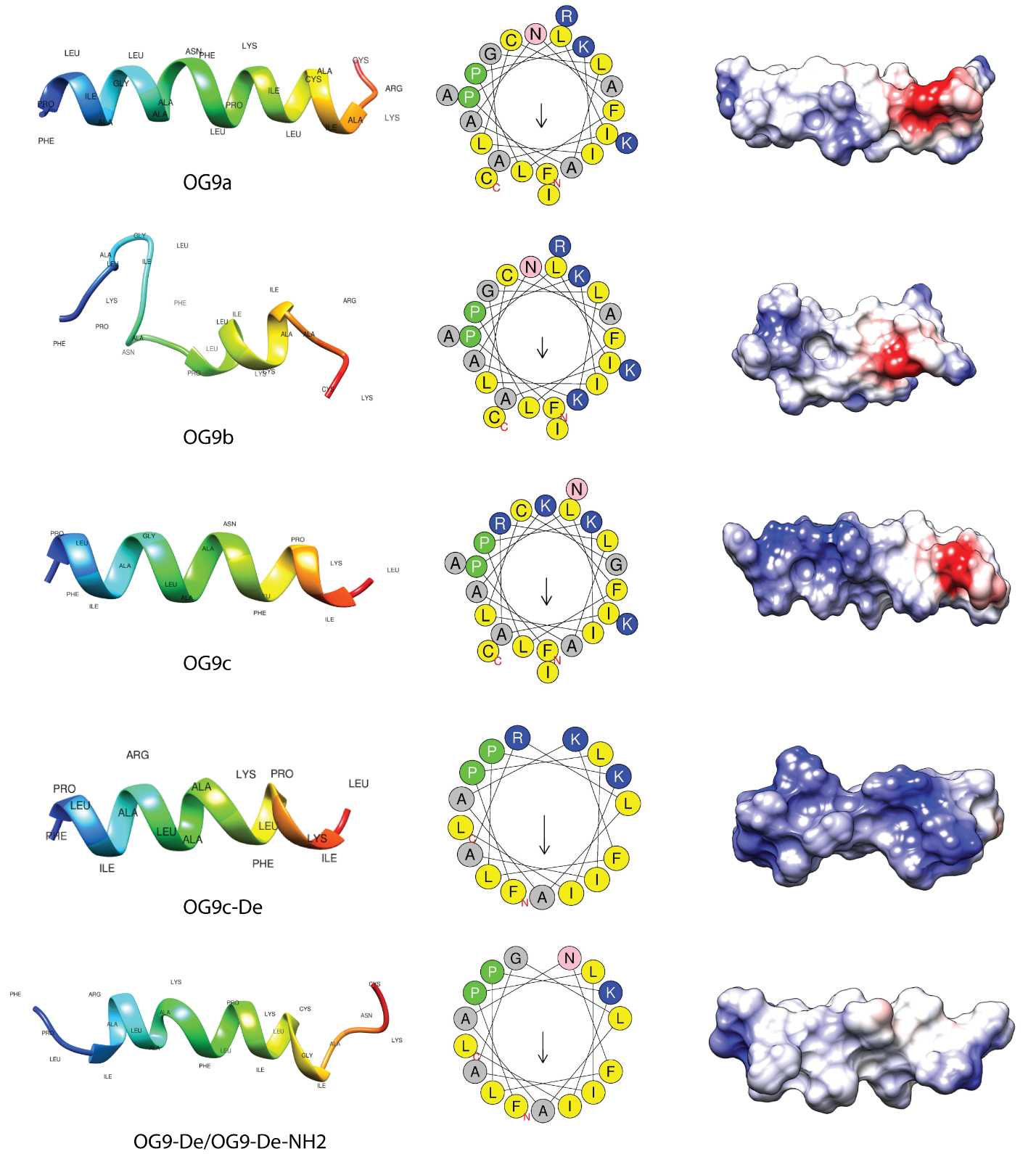


**Figure S2.** Three-dimensional models, helical wheel diagrams, and electrostatic potential surface diagrams of OG9a, OG9b, OG9c, OG9c-De, OG9-De, and OG9-De-NH_2_.

**Table S1.** MBICs/ MBECs (μM) of OG9 and OG9c-De-NH_2_ against selected pathogens:

| Org. | Gram positive (+) | | | | | Gram negative (-) | | |
| --- | --- | --- | --- | --- | --- | --- | --- | --- |
| Name | *S. aureus* ATCC 6538 | | MRSA NCTC 12493 | *E. faecalis* NCTC 12697 | *E. coli* ATCC 8739 | | *K. pneumoniae* ATCC 43816 | *P. aeruginosa* ATCC 9027 |
| OG9 | 16/>128 | 16/>128 | | 32/>128 | | 32/>128 | >128/>128 | >128/>128 |
| OG9c-De-NH_2_ | 1/>128 | 1/128 | | 16/>128 | | 16/>128 | >128/>128 | 64/>128 |


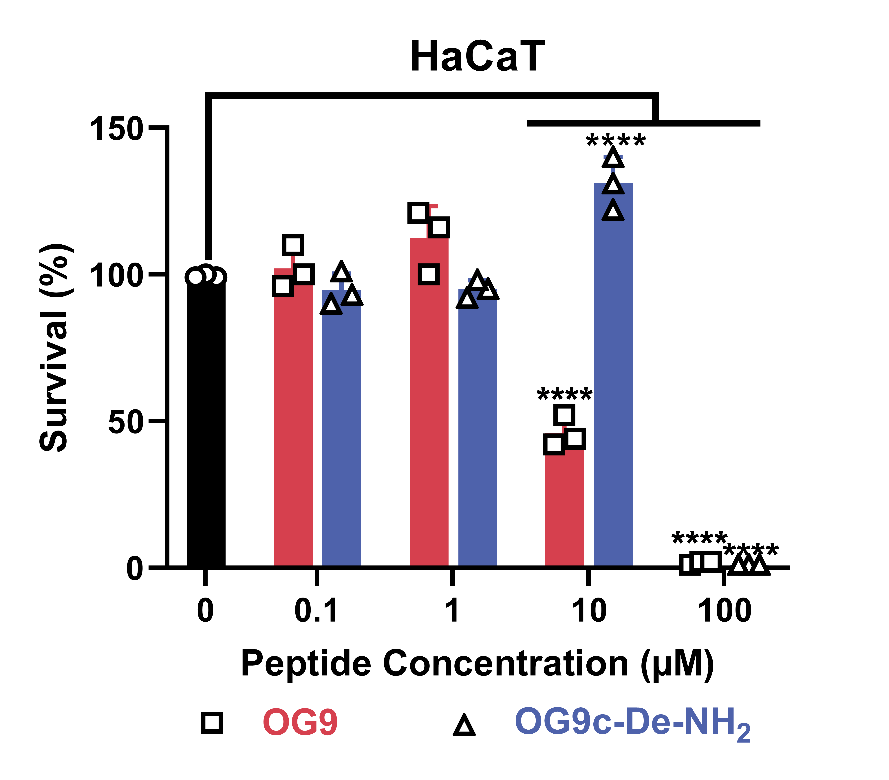


**Figure S3.** Cytotoxicity of OG9 and OG9c-De-NH_2_ against human keratinocytes HaCaT cells. The data are derived from three independent experiments and shown as the means ± SEM. The significance is indicated by **** (p < 0.0001).
